# Preparing of Point-of-Care Reagents for Risk Assessment in the Elderly at Home by a Home-Visit Nurse and Verification of Their Analytical Accuracy

**DOI:** 10.3390/diagnostics13142407

**Published:** 2023-07-19

**Authors:** Shoji Takenaka, Hiroshi Moro, Utako Shimizu, Takeshi Koizumi, Kei Nagano, Naoki Edanami, Naoto Ohkura, Hisanori Domon, Yutaka Terao, Yuichiro Noiri

**Affiliations:** 1 Division of Cariology, Operative Dentistry and Endodontics, Niigata University Graduate School of Medical and Dental Sciences, Niigata 951-8514, Japan; 2 Department of Respiratory Medicine and Infectious Diseases, Niigata University Graduate School of Medical and Dental Sciences, Niigata 951-8514, Japan; 3 Faculty of Medicine, Niigata University Graduate School of Health Sciences, Niigata 951-8514, Japan; 4 Division of Microbiology and Infectious Diseases, Niigata University Graduate School of Medical and Dental Sciences, Niigata 951-8514, Japan

**Keywords:** pneumonia, point-of-care testing, C-reactive protein, white blood cells, neutrophil-to-lymphocyte ratio

## Abstract

With the rising number of older adults residing at home, there is a growing need for risk assessment and patient management in home nursing. This study aims to develop point-of-care test (POCT) reagents that can aid in risk assessment and home care, especially in settings with limited resources. Our focus was on creating a C-reactive protein (CRP) POCT, which can accurately diagnose clinically significant judgment values in home nursing. Additionally, we assessed the utility of the HemoCue WBC DIFF system in providing differential counts of white blood cells (WBC). These performances were compared with a laboratory test using blood samples from patients with pneumonia. The CRP POCT showed a comparable result to that of a laboratory method, with an average kappa index of 0.883. The leukocyte count showed good agreement with the reference method. While the correlation coefficients for both neutrophil and lymphocyte counts were deemed acceptable, it was observed that the measured values tended to be smaller in cases where the cell count was higher. This proportional error indicates a weak correlation with the neutrophil-to-lymphocyte ratio. CRP POCT and WBC counts provided reliable and accurate judgments. These tools may benefit risk management for older adults at home, patients with dementia who cannot communicate, and those living in depopulated areas.

## 1. Introduction

The population of older people is increasing worldwide. The share of the global population aged 65 years or above is projected to rise from 10% in 2022 to 16% in 2050 [1]. Notably, Japan has the highest percentage of the total population aged 65 years old and over, reaching 28.6% in 2020, and exceeding the U.S.A. (16.6%), Sweden (20.3%), France (20.8%), Germany (21.7%), and Italy (23.3%) [2]. The aging society in Japan is progressing quite rapidly compared to the U.S.A. and European countries. The Japanese government proposed establishing a community-based integrated care system by 2025 to comprehensively ensure the provision of healthcare, nursing care, preventive care, housing, and livelihood support to the older population to solve this social problem [3]. The objective of this system is to promote the independent living of older adults within their familiar community for as long as possible, ensuring their dignity is preserved and providing support for their autonomy. To achieve this goal, a visiting nurse needs to detect changes in the physical condition of older adults and decide whether it is better to take them to a hospital or to give them antibiotics and monitor their progress. However, because the equipment that can be brought in for home care is limited, visiting nurses have no choice but to perceive the risk through experience based on limited physiological parameters, such as heart rate, systolic blood pressure, respiratory rate, oxygen saturation, level of consciousness, and temperature. Prehospital early warning scores (EWS) help to identify patients at risk of deterioration who need a referral to secondary care [4,5]. Recently, EWS was also implemented in health management at a nursing home [6,7]. However, the older population often presents with nonspecific clinical symptoms and functional decline, which renders accurate diagnosis difficult and may lead to a life-threatening delay in therapy [8]. For instance, the fever response is often blunted even in the presence of bacteremia [8].

Point-of-care tests (POCTs) enable early detection of acute infections and diseases such as cancer, diabetes, and cardiovascular diseases [9,10]. As POCT also contributes to the monitoring of health conditions [11], its use is widespread across a variety of settings, including intensive care units, emergency departments, operating rooms, general practitioners, nursing homes, and inpatient care [12]. Among the many currently available diagnostic POCTs [13], we focused on C-reactive protein (CRP) levels and differential white blood cell (WBC) counts. CRP and WBC counts are often used as nonspecific markers of bacteremia and invasive local infections [14]. CRP is an acute-phase protein synthesized by the liver in response to the secretion of several inflammatory cytokines, including interleukin (IL)-6, IL-1, and tumor necrosis factor [15], and its serum concentration rises above 5 mg/L within 6 h, reaching a peak at 48 h, with a half-life of approximately 19 h [16]. As CRP levels reflect ongoing inflammation and/or tissue damage in various diseases, the cause of inflammation cannot be identified. However, CRP is an essential test in clinical laboratories when infection is suspected [15,16]. Furthermore, the neutrophil-to-lymphocyte ratio (NLR) is a reliable marker for diagnosing bacteremia and diseases such as sepsis, pneumonia, and cancer [17]. Some investigations have demonstrated that serial NLR is useful for predicting the prognosis and early treatment response of hospitalized community-acquired pneumonia (CAP) [18,19]. Thus, we speculated that a combination of POCT of CRP and NLR would assist in risk management for older patients at home. Several studies have been performed to test the efficacy of POCT in nursing homes [20,21], but the types of POCT reagents that are useful for risk assessment of older patients at home, their effectiveness, and their standard values remain unclear.

**Figure 1:**
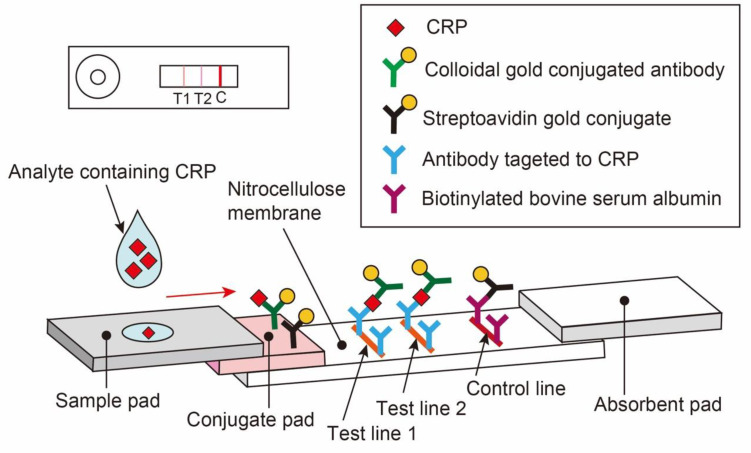
Schematic representation of the CRP POCT developed in this study. Colloidal gold conjugated antibody: Anti-human CRP (Anti-h CRP 6407 SPTN-5, Medix Biochemica, Espoo, Finland)-sensitized colloidal gold (gold colloid solution SC, Tanaka Kikinzoku Kogyo K.K., Tokyo, Japan). Streptavidin gold conjugate: Streptoavidin (Prospec-Tany technogene Ltd., Rehovot, Israel) -sensitized colloidal gold. Antibody targeted to CRP: Anti-human CRP antibody (Goat anti-human CRP antibody, A80-125A, Bethyl Laboratories, Montgomery, TX, USA). Biotinylated BSA: Biotin NHS-water soluble, VSP-1210-50, Vector Laboratories, Newark, CA, USA; BSA, A7030, Sigma-Aldrich Japan, Tokyo, Japan. T1: Test line 1, T2: Test line 2, C: Control line. CRP, C-reactive protein; POCTs, point-of-care tests; BSA, bovine serum albumin.

**Figure 2:**
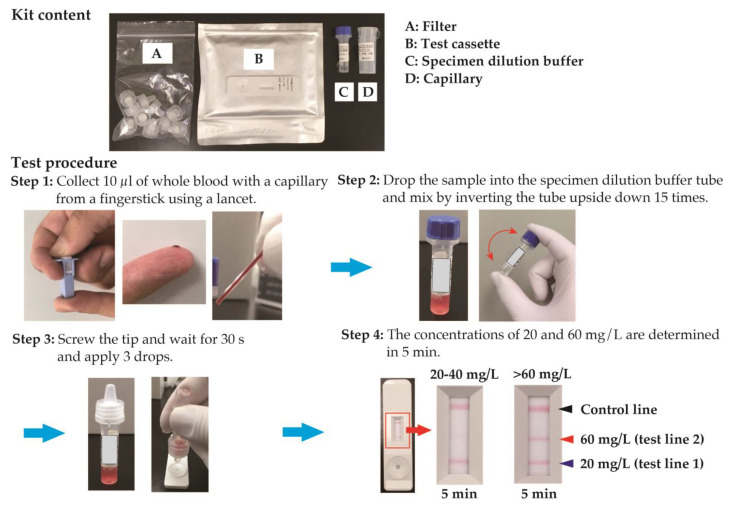
Kit content and test procedure of HC-CRP. A K2-EDTA whole blood sample was utilized instead of peripheral blood (Step 1). Following this, 10 µL of whole blood was added to the dilution buffer (Step 2). The subsequent step involved the use of a filter to remove blood cell components (Step 3). Test lines 1 and 2 were coated with anti-human CRP antibody at two different concentrations, while the control line was coated with biotinylated BSA (Step 4). The absence of a test line indicates a CRP concentration below 20 mg/L. When only test line 1 is visible, the CRP concentration falls within the range of 20–60 mg/L. Conversely, the appearance of both test lines (1 and 2) signifies a CRP concentration exceeding 60 mg/L.

This study aims to prepare POCT reagents for detecting the risk of bacterial infections in older adults at home, where resources are limited, and to examine analytical accuracy. We have developed a semiquantitative rapid test cassette for CRP level determination in whole blood samples, with a scientific basis and a threshold value considered effective for risk assessment of older adults at home. This reagent is based on the principle of a colloidal gold immunochromatographic assay, which was provided by Hokudo Co., Ltd., Sapporo, Japan (HC-CRP; Figure 1). The test results were read visually without using any instrument. This device provides two lines of qualitative detections of 2 and 6 mg/L CRP after 5 min, after collecting 10 µL of whole blood from a fingertip using a lancet (Figure 2). A list of equipment, reagents, and methods used in this study is presented in Supplementary Materials (Files S1 and S2).

A retrospective cohort study demonstrated that a CRP level of 60 mg/L was a predictive cutoff value for pneumonia, showing that a CRP level of >60 mg/L was independently associated with a 3.59-fold increased risk of pneumonia [22]. In another cluster randomized controlled trial, physicians increasingly prescribed antibiotics for patients with CRP levels >40 mg/L and almost always prescribed antibiotics when CRP levels were >60 mg/L, based on the available evidence and the current Dutch guideline recommendations on lower respiratory tract infections [23,24]. Recent systematic reviews have shown that using CRP POCT as an adjunct to standard care reduces the number of antibiotic prescriptions in primary care patients with acute respiratory infection symptoms [25,26,27]. Furthermore, antibiotics are unlikely to be beneficial and should not be prescribed for patients with a CRP level lower than 20 mg/L [28,29]. Based on these reports, the CRP levels were set at 20 and 60 mg/L.

The NLR was determined using a WBC differential counter (HemoCue WBC DIFF system, HemoCue AB, Ängelholm, Sweden), and the accuracy was evaluated by comparing it with the quantitative value of a routine laboratory test.

## 2. Materials and Methods

### 2.1. Study Population and Sampling

Thirty-one patients with CAP admitted to Niigata University Medical and Dental Hospital (Niigata, Japan) were included in the study between February 2020 and February 2022. Clinical diagnosis of pneumonia was defined as a new radiographic infiltrate compatible with pneumonia, the presence of a lower respiratory tract infection, the presence of at least one episode of fever (>38 °C), leukocytosis (>11.0 × 10^9^/L), leukopenia (<3.5 × 10^9^/L), purulent sputum, a change in the character of respiratory secretions, and/or an increased arterial-alveolar gradient [30]. Hospital admission was determined when the A-DROP score was greater than 2 [31]. The patients were managed according to the American Thoracic Society guidelines [32]. The study protocol was approved by the Niigata University Ethics Committee (approval number 2020-0006) and was conducted in accordance with the approved guidelines. All the patients signed an informed consent form before participating in the study.

A K_2_-EDTA whole blood sample collected for a therapeutically necessary blood test during hospitalization was subjected to testing. Sampling was performed until the patient was cured, transferred to another hospital, or died. The Supplementary Materials (File S3) summarizes the patients’ characteristics.

### 2.2. Measurement of CRP Level

The determination of CRP levels by HC-CRP and Actim CRP (Medix Biochemica, Kauniainen, Finland) were compared with a standard laboratory test (TBA-2000FR, Canon Medical Systems Corp., Tochigi, Japan). Actim CRP, a rapid dipstick test based on monoclonal antibodies, has three different detection limits: 10–40, 40–80, and >80 mg/L Supplementary Materials (File S4) [33]. CRP POCTs do not require any laboratory measuring instruments or power supplies. A total of 169 K_2_-EDTA blood samples, which ranged from 5–100 mg/L CRP measured using the peripheral blood with a TBA-2000FR, were used for CRP POCT determination. The measured values were evaluated by two physicians who were blinded to the quantitative values.

### 2.3. WBC Differential Counting

Five-part differential counting of WBC in 68 blood samples was performed within 30 min of sampling using the HemoCue WBC DIFF. The values were compared with those obtained by clinical examination (XR-9000; Sysmenx Corp., Hyogo, Japan). The HemoCue WBC DIFF provides counts of neutrophils, lymphocytes, monocytes, eosinophils, and basophils within 5 min. The principle is based on recognizing white cells stained with methylene blue [34]. This analyzer operates on batteries, so a power supply is not always required. The measurement range was between 0.3–30.0 × 10^9^/L.

### 2.4. Statistical Analyses

The accuracy between CRP POCT and quantitative values of laboratory tests and intra-evaluator agreement was calculated using the kappa index (SPSS version 29, IBM Corp., Armonk, NY, USA). Passing–Bablok correlation analysis was conducted to evaluate the correlation between the HemoCue WBC DIFF and hospital examinations (XLSTAT ver. 2023.1.2, Lumivero, Paris, France). The Bland–Altman method was used to evaluate bias and trends (SPSS version 29). Systematic errors were analyzed using a paired t-test for fixed errors and a linear regression analysis for proportional errors (SPSS version 29). Statistical significance was set at *p* < 0.05.

## 3. Results

### 3.1. Analytical Accuracy between CRP POCT and Clinical Examination

**Figure 3:**
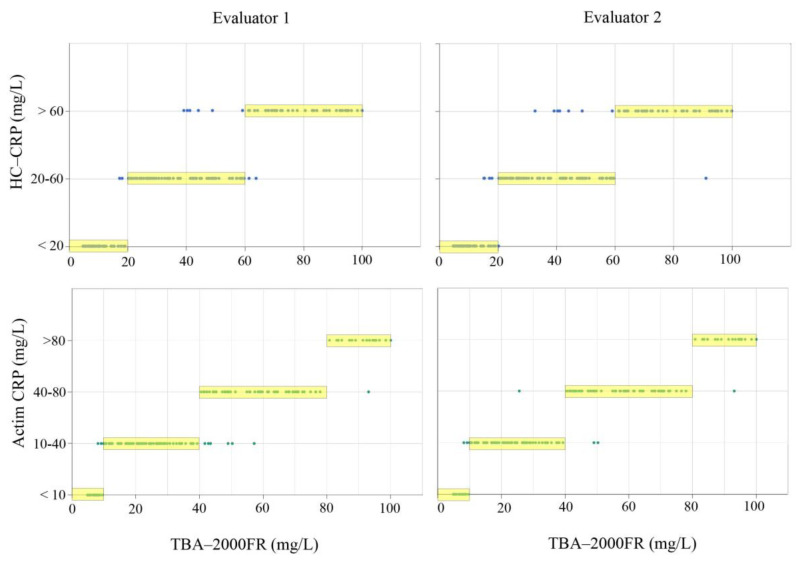
Correlation of CRP POCT with clinical examination. Each dot represents one sample (*n* = 169). The X and Y axes represent the quantitative value obtained from a laboratory test and the qualitative value obtained from HC-CRP, respectively. The corresponding values were plotted at the appropriate intersection. The yellow boxes represent the corresponding categories on each test. CRP, C-reactive protein; POCT, point-of-care test.

The correlation between the CRP POCTs and clinical examination results is shown in Figure 3. The HC-CRP kappa index compared with TBA-2000FR indicated excellent agreement between the evaluators and raters (Table 1). However, the two raters’ agreements in the 20–60 mg/L detection range differed. This is due to the fact that the test strip was visually judged, meaning that the accuracy varies depending on the evaluator. The kappa index for Actim CRP and TBA-2000FR showed almost complete agreement (Figure 3, Table 1). Inter-evaluator ratings were also consistent. Actim CRP levels showed a high agreement of coincidence across all detection ranges.

### 3.2. Analytical Accuracy between POCT and Clinical Examination of WBC Differential Counting

**Figure 4:**
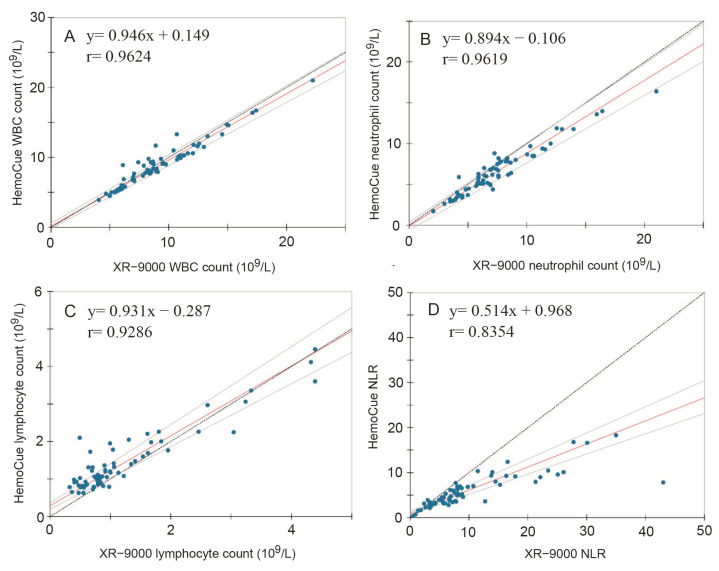
Passing−Bablok correlation of (**A**) WBCs, (**B**) neutrophils, (**C**) lymphocytes, and (**D**) NLR. WBC, white blood cell; NLR, neutrophil-to-lymphocyte ratio; r, correlation coefficient. The solid red line represents the regression line. The bold dashed line represents the identity line. The thin black line represents the confidence interval for the regression line.

**Figure 5:**
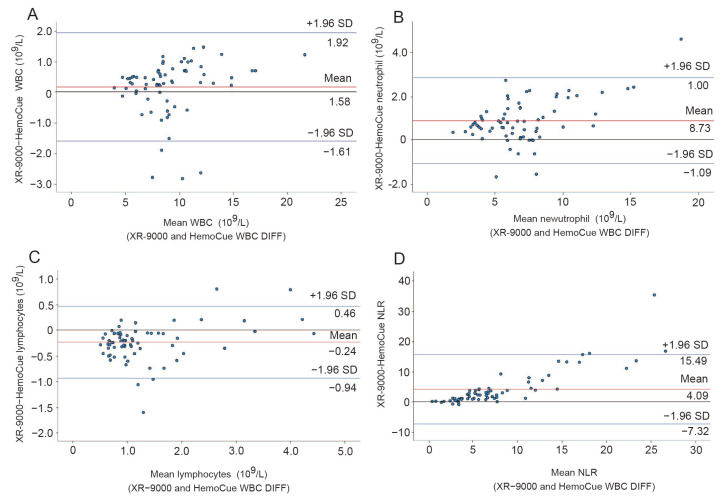
Bland–Altmann plots with a mean count of total (**A**) WBC, (**B**) neutrophils, (**C**) lymphocytes, and (**D**) NLR plotted against the differences between the two methods with mean bias and 95% limit of agreement. WBC, white blood cell; NLR, neutrophil-to-lymphocyte ratio. The solid red line represents the average difference between measurements of XR-9000 and the HemoCue WBC DIFF system. The black line indicates zero. Blue lines indicate the limits of agreement.

The consistency between the HemoCue WBC DIFF system results and the XR-9000 test results was compared in this study. The value ranges were as follows: 4.03–22.21 × 10^9^/L for WBC, 2.04–20.99 × 10^9^/L for neutrophils, 0.323–4.392 × 10^9^/L for lymphocytes, and 0.46–42.96 for NLR according to the XR-9000. The Passing–Bablok correlation between WBCs, neutrophils, lymphocytes, and NLR is shown in Figure 4. WBC count showed the highest correlation coefficient (r = 0.9624) between the HemoCue WBC DIFF system and XR-9000, with slope and intercept of 0.946 (95% confidence interval [Cl]: 0.901–0.983) and 0.149 (95% Cl: −0.196–0.559), respectively (Figure 4A). Bland–Altman plots with mean counts of total WBC, neutrophils, and lymphocytes for the two methods are shown in Figure 5. The bias found in the comparison between the two instruments was 1.58 × 10^9^/L (Cl: −1.61–1.92 × 10^9^/L) (Figure 5A). Although the four paired samples plotted out the lowelimit of agreement, no systematic error was detected (*p* = 0.154 for fixed error and *p* = 0.119 for proportional error).

The correlation for the neutrophil count was r = 0.962, with a slope of 0.894 (95% Cl: 0.827–0.973) and an intercept of −0.106 (95% Cl: −0.604–0.348), respectively (Figure 4B). The bias found in the comparison between the two instruments was 8.73 × 10^9^/L (95% Cl: −1.09–1.00 × 10^9^/L) (Figure 5B). A comparison of the three paired samples was plotted over the limits of agreement. Fixed (*p* < 0.001) and proportional (*p* < 0.001) errors were also detected. As the neutrophil count increased, the difference between the HemoCue WBC DIFF system and the XR-9000 tended to increase.

The HemoCue WBC DIFF lymphocyte count also displayed a good correlation (r = 0.9286) with XR-9000, with a slope of 0.931 (95% Cl: 0.838–1.042) and an intercept of −0.287 (95% Cl: 0.186–0.366) (Figure 4C). However, four paired samples plotted outside the acceptable range and systematic errors were detected (*p* < 0.001 for fixed errors and *p* = 0.003 for proportional errors). The bias found in the comparison between the two instruments was −0.24 × 10^9^/L (95% Cl: −0.94–0.46 × 10^9^/L) (Figure 5C). As the lymphocyte and neutrophil count increased, the discrepancy between the two measurements increased.

The NLR showed an acceptable correlation (r = 0.8354); however, the slope coefficient was well below a proportional difference of 1 (0.514, 95% Cl: 0.449–0.582). The intercept also deviated from zero, and the confidence interval did not include zero (0.968, 95% Cl: 0.665–1.363) (Figure 4D). The bias found in the comparison between the two instruments was 4.09 (95% Cl: −7.32–15.49) (Figure 5D). The model coefficient revealed both fixed and proportional errors (*p* < 0.001), showing that the NLR and the difference between the two measurements increased (Figure 5D).

## 4. Discussion

Japan is the first super-aging society in the world, with one in three people being elderly [3]. The prevalence of dementia in people older than 65 years is projected to exceed 25% nationwide, including in the metropolitan areas [35]. The Japanese government, academia, and industry are working together to implement various initiatives, including community-based comprehensive care [3]. The Japanese government has recommended that older adults reside in their homes, enabling them to maintain their daily routines and receive support and nursing services through visits from care staff or nursing-care facilities [36]. In such cases, medical care is provided by home-visit nurses under the guidance of physicians, allowing service users to continue living at home. However, in areas with a low population or a lack of doctors, it can be time consuming for older adults to seek medical attention when there is an increased risk of bacterial infections. To address this issue and support home-visit nurses in detecting the risk of bacterial infections among elderly individuals at home, we have developed a CRP POCT that does not require a dedicated measuring device or power source. This enables home-visit nurses to collaborate remotely with doctors in determining whether the elderly individual needs to visit a hospital or receive antibiotics. The development of POCT reagents for assessing the risk of bacterial infections in elderly individuals who face challenges in visiting hospitals holds potential applicability in telemedicine and disaster medicine.

HC-CRP, the first reagent of its kind in Japan, eliminates the need for a dedicated measuring device or power supply and allows for visual determination. We have set scientifically meaningful thresholds of 20 mg/L for home care purposes. While numerous CRP immunochromatographic reagents are available worldwide [33,37], most products utilize thresholds of 10, 40, and 80 mg/L. Although these thresholds may prove useful in primary care settings, they may not be as applicable in the context of home care.

Antimicrobial resistance is a global problem driven by the misuse and abuse of antibiotics. The utility of POCT, including CRP, as a strategy to safely reduce antibiotic prescriptions is being debated among experts [38]. CRP POCT, as an adjunct to standard care, likely reduces the number of participants given an antibiotic prescription among primary care patients presenting with acute respiratory infection symptoms [25]. In a randomized controlled trial, Boere et al. evaluated whether CRP POCT safely reduced antibiotic prescriptions in 241 nursing home residents with suspected lower respiratory tract infections [24]. By utilizing CRP, the prescribing rate of antibiotics decreased by 28.8% (odds ratio [OR] = 4.93, 95% Cl: 1.91–12.73) without significant effect on full recovery, mortality, and hospital admission rate. Van den Bruel et al. systematically reviewed the diagnostic value of serious infections in febrile children in an ambulatory setting. They reported that 80 mg/L CRP is recommended to rule in serious infections, and 20 mg/L CRP is necessary to rule out serious infections [39]. Butler et al. conducted a multicenter, open-label, randomized controlled trial involving primary care patients diagnosed with chronic obstructive pulmonary disease. The patients were randomly assigned to either receive usual care guided by CRP POCT or usual care alone. The results demonstrated that fewer patients in the CRP-guided group, with CRP values lower than 20 mg/L, reported antibiotic use compared to the usual care group (32.8% vs. 77.4%), without compromising recovery, hospitalization, and mortality after three weeks [28]. Therefore, the threshold for test line 1 was set at 20 mg/L. The use of CRP POCT with a threshold of 20 mg/L for diagnosis may help reduce unnecessary antibiotic administration, thereby potentially reducing medical costs and suppressing drug-resistant bacteria. In other words, achieving appropriate antimicrobial use holds significant importance.

The threshold of 60 mg/L in HC-CRP (test line 2) may not hold substantial significance in home medical care because the person will be taken to the hospital. However, the value may be useful to imagine whether pneumonia is associated with bacterial infection or with viral infection alone. Korppi et al. demonstrated that serum CRP over 60 mg/L separated cases with evidence of pneumococcal aetiology from those with proved pure viral aetiology with a sensitivity of 0.26 and specificity of 0.83 [40].

In addition, by simultaneously measuring both WBC and CRP, it may aid in identifying the timing of infection, early determination of therapeutic effects, and assessing the severity of the disease. CRP levels do not significantly rise during the early stages of infection but increase with disease progression. The peak of CRP does not coincide with that of WBC [41]. Neutrophils, as the first inflammatory cells to arrive at the site of inflammation prior to CRP production, play a role in bacteria phagocytosis and release chemotactic mediators that recruit other leukocytes to the affected tissue [42]. If only CRP levels are elevated, it can be inferred that the infection is in the later stages. Although data on the diagnostic accuracy of CRP in differentiating infection from non-infection are inconclusive, simultaneous measurement of WBC and CRP provides moderate discrimination [43]. Kapasi et al. conducted a review on the diagnostic performance of host biomarkers for differentiating bacterial from non-bacterial infections to guide antibiotic use [44]. Among the 193 identified citations, 59 studies evaluating over 112 host biomarkers were selected. The most frequently evaluated host biomarkers were CRP (61%), white blood cell count (44%), and procalcitonin (34%). Overall, we believe that HC-CRP and WBC counting serve as useful diagnostic, prognostic, and monitoring tools for bacterial infections in home care settings.

In this study, we examined the analytical performance characteristics and usefulness of the HemoCue WBC DIFF counter in patients with pneumonia. The most reliable measurement was the leukocyte count, which showed a correlation coefficient, slope, and intercept close to 1 without any systematic error (Figure 4A). Although the neutrophil and lymphocyte counts showed acceptable correlation coefficients, proportional errors were included. The higher the cell number, the smaller the value measured by HemoCue WBC DIFF compared to that of a laboratory test (XR-9000). The number of neutrophils and lymphocytes was very high in this study because the subjects were patients with acute pneumonia who required hospitalization. Neutrophil and lymphocyte measurements may be unreliable if presented with excessively large values. These findings are consistent with a previous report by Mattsson et al., who conducted a prospective feasibility and measurement study comparing HemoCue WBC DIFF with standard measurements in cancer patients [45]. Weighted Deming regression analysis showed significant proportional bias between methods, showing that the values measured by HemoCue tended to be underestimated compared to the standard method, as the total WBC and neutrophils counts increased. In this study, the slope of NLR was only 0.514 because of the proportional error of both neutrophils and lymphocytes (Figure 4D). If the analysis was performed using data with an NLR of 10 or less, the slope coefficient increased to 0.703. These results suggest that NLR may be underestimated if the neutrophil count is abnormally high. Cataudella et al. conducted a prospective clinical study to explore the performance of NLR in a cohort of elderly adults with CAP. These results suggest that short-term in-hospital care is needed for those with an NLR greater than 11.12, and admission to a respiratory intensive care unit is needed when the NLR is greater than 28.3 [19]. When using the HemoCue WBC DIFF to measure NLR in home care, paying attention to the possibility of underestimation is necessary. Although some studies reported that Hemocue devices are comparable to the clinical laboratory analyzer for WBC [34,45,46,47,48], neutrophil [34,46,47], and lymphocyte counts [46,47], no studies have reported the reliability of NLR.

This study has several limitations. First, all analyses were performed using whole blood rather than peripheral blood from the fingertip because of the critical illness of the subjects. Consequently, there is no data on the correlation between the capillary samples obtained using POCT devices and the value of venous samples in a routine laboratory test. The reliability of the POCT when used in home care has not yet been proven. In a previous report, leukocyte counts in capillary and venous blood measured using HemoCue WBC showed no significant differences, although the number of subjects was limited [49]. Another limitation is that its usefulness in home medical care remains unknown. Although this study demonstrated the reliability of the POCT device for the rapid diagnosis of CRP and WBC, whether these POCTs can improve the ability to identify patients at risk of deterioration remains unknown.

HC-CRP has high accuracy compared to standard laboratory tests in patients with CAP. A high kappa coefficient was identified, which was considered reproducible across all detection ranges (Figure 3, Table 1). However, there were variations in the agreements between the two raters within the detection range of 20–60 mg/L. This discrepancy is a limitation of the immunochromatographic reagent, as the visual determination can differ depending on the evaluator. Similar to this study, previous studies have also assessed the analytical performance of commercially available CRP POCTs based on immunochromatographic assay by two evaluators [33,37]. However, it seems necessary to compare the judgments of more raters for correct performance evaluation, given the variability among evaluators. There are also several potential disadvantages of the immunochromatographic assay such as low sensitivity, limited precision quantification ability, and no pathogen identification [50,51,52,53]. Immunoassay kits may not pick up on low levels of the analyte, leading to false negatives, or it might give a positive result where none should exist, leading to false positives. Overall, the use of immunoassay kits in POCT is a balance between the need for quick, actionable results and the issues related to cost, quality, and accuracy. Since visual judgment with the naked eye can introduce ambiguity in the criteria, devices that combine microfluidic POCT and colorimetric sensors coupled with smartphones are currently under development [10,53]. This study was the first step toward establishing a scientific assessment method for visiting nurses to detect the risk of developing infectious diseases in the elderly. It will be necessary to combine multiple biomarkers and develop a POCT device using an electron optical system to overcome the disadvantages of the immunochromatographic assay.

Additionally, it should be noted that HC-CRP and HemoCue WBC DIFF counters are currently approved for use only by physicians and nurses. Even if these devices offer acceptable analytical quality and are well-received by older adults in home settings, significant regulatory challenges need to be addressed and resolved. These challenges encompass medical significance, efficacy, and cost-effectiveness, which must be tackled before this technology can be implemented in routine healthcare settings.

## 5. Conclusions

This study provided reliable and accurate comparative results between two types of CRP POCTs and WBC counts using the HemoCue WBC DIFF. These findings may benefit risk management for older adults at home, patients with dementia who cannot communicate, and those living in depopulated areas. Further investigations are needed to evaluate the impact of various POCTs on decision making in home nursing and their cost-effectiveness.

## Figures and Tables

**Table 1:** Correlation between CRP POCT and clinical examination. The correlation is expressed with the kappa index and the degree of coincidence. CRP, C-reactive protein; POCT, point-of-care test.

	Targets for Comparison	Kappa Index	Degree of Coincidence (%)			
Detection range (mg/L)			<20	20–60	>60	
HC-CRP	TBA-2000FR vs. evaluator 1	0.910	96.6	91.4	95.1	
	TBA-2000FR vs. evaluator 2	0.856	93.1	84.2	97.6	
	Evaluator 1 vs. evaluator 2	0.910				
Detection range (mg/L)			<10	10–40	40–80	>80
Actim CRP	TBA-2000FR vs. evaluator 1	0.916	96.6	96.9	98.2	100
	TBA-2000FR vs. evaluator 2	0.932	89.7	96.9	94.5	100
	Evaluator 1 vs. evaluator 2	0.881				

## Data Availability

Data presented in this study are available upon reasonable request from the corresponding author owing to restrictions such as privacy or ethical concerns. The data are not publicly available because of the assured participant confidentiality.

## Supplementary Materials

The following supporting information can be downloaded from: https://www.mdpi.com/article/10.3390/diagnostics13142407/s1, File S1: List of equipment and reagents used; File S2: Methods of a reagent preparation; File S3: The characteristics of patients; File S4: Representative images of Actim CRP at each detection range.
