# Supplementary material for: Preparing of Point-of-Care Reagents for Risk Assessment in the Elderly at Home by a Home-Visit Nurse and Verification of Their Analytical Accuracy
Source: Diagnostics (Basel). 2023 Jul 19;13(14):2407. doi: 10.3390/diagnostics13142407 (PMC10378029; doi:10.3390/diagnostics13142407)
Supplement: Supplementary file 1 [file diagnostics-13-02407-s001.zip › Supplementary File S1.pdf]

File S1. List of equipment and reagents used.

Product Name	Company	Catalogue number	Lot number
Microvolume spectrophotometer	DeNovix Inc., DE, U.S.A.	DS-11+	
Ultrasonic cleaning machine	Sharp Corporation, Osaka, Japan	UT-106	
Cutting module	BioDot Inc., CA. U.S.A.	CM4000	
Antibody coating machine		PD1020	
Vacuum desiccator	AS ONE Corp., Osaka, Japan	Type VW	
Dry vacuum pump	ULVAC, Inc., Kanagawa, Japan	DA-60D	
Heater incubator	PHC Corp., Ehime, Japan	MIR-H263-PJ	
Electronic balance	A&D Company, Ltd., Tokyo, Japan	FA-200	
Analytical balance		HR-60	
Micro cooled centrifuge	TOMY Digital Biology Co., Ltd., Tokyo, Japan	MX-307	
PH meter	Mettler-Toledo International, Inc., OH, U.S.A.	Seven easy	
Low protein adsorption tube	FUKAE KASEI CO., Ltd., Hyogo, Japan	PK-20C-500	
Nitrocellulose membrane (Hi-Flow Plus HFC13502)	Merck Millipore, MA, U.S.A.	HF13502XSS	R0JB99676C
Sample absorbent pad (Cellulose Fiber Sample Pad)		CFSP223000	
Glass fiber conjugate pad (GFCP)		GFCP103000	92065001
Plasma separator pad (CytoSep Grade 1660)	PALL Corp., NY, U.S.A.	S70002	T12591
Backing sheet	NIPPN engineering Co., Ltd., Tokyo, Japan	334042/11GL-56338	070158-01
Housing case	NIPPN engineering Co., Ltd., Tokyo, Japan	32208/300	

Filter	Vessel Co. Ltd., Tokyo, Japan		
Endo to endo pipettes	AS ONE Corp., Osaka, Japan	3-5998-07	2411528
Anti-hCRP 6407 SPTN-7	Medix Biochemica, Espoo, Finland	100147	0044968
Goat x-Human C-Reactive protein	Bethyl Laboratories, Texas, USA	A80-125A	11
AU colloidal solution-SC (particle size 40nm)	TANAKA Holdings Co., Ltd., Tokyo, Japan		A201016-1F
BIOTIN NHS-water Soluble	Vector Laboratories, California, USA	SP-1210	ZE0316
Streptavidin	Prospec-Tany technogene Ltd., Rehovot, Israel	Pro-791	918PSTREP
Silica gel	Tokai chemical industry Co., Ltd., Aichi, Japan		81102
BSA	Sigma-Aldrich Japan, Tokyo, Japan	A3050-50G	SLBT8252
Trizma base		T1503-1KG	SLBW6320
ProClin300		48912 U	MKCK5149
Potassium chloride	FUJIFILM Wako Pure Chemical Corp., Tokyo, Japan	163-03545	WDQ2822
Sucrose		196-00015	CAH4164
Polyethylene glycol 20,000		168-11285	SDN0465
Sodium azide		197-11091	MCG7769
Casein		030-01505	PTK2231
Sodium hydrogen carbonate		191-01305	PTQ-3293
Sodium cholate		197-08502	KWE2070
Sodium dodecyl sulfate		191-07145	CDN8564
Glycine	KANTO Chemical Co., Inc., Tokyo, Japan	01194-00	211K1374
Sodium chloride		37144-00	012C5603
Hydrochloric acid		18078-00	012H1018
Sodium hydroxide		37184-00	010H6096
Boric acid		04232-00	910H1296
Sodium carbonate		37141-00	707X2043
Disodium hydrogen		37240-00	008H1536

phosphate			
Potassium dihydrogen phosphate		32379-00	003H1254
Sodium dihydrogen phosphate dihydrate		37239-00	909H1719
