# Supplementary material for: Preparing of Point-of-Care Reagents for Risk Assessment in the Elderly at Home by a Home-Visit Nurse and Verification of Their Analytical Accuracy
Source: Diagnostics (Basel). 2023 Jul 19;13(14):2407. doi: 10.3390/diagnostics13142407 (PMC10378029; doi:10.3390/diagnostics13142407)
Supplement: Supplementary file 1 [file diagnostics-13-02407-s001.zip › Supplementary File S2.pdf]

File S2. Methods of a reagent preparation.

1) Preparations for Anti-human CRP (Anti-h CRP 6407 SPTN-5)-sensitized colloidal gold and Streptoavidin-sensitized colloidal gold.

a) Anti-human CRP was adjusted to 100 μg/ml after substitution with phosphate buffer.
b) Add 100 μl of potassium dihydrogen phosphate buffer to a 2.0 ml tube.
c) 900 μl of colloidal gold solution was added to the above tube and gently stirred.
d) Add 100 μl of the antibody solution (100 μg/ml) prepared in a) to the above tube while stirring, and incubate at room temperature for 10 min,
e) 55 μl of polyethylene glycol solution was added to the above tube and gently stirred.
f) 110 μl of BSA solution was added to the above tube and gently stirred.
g) The tube was centrifuged at 8,000g for 15 min at 4ºC.
h) The supernatant was removed except for about 100 μl, and the precipitate was dispersed in the remaining supernatant for 2 min.
i) 1.5 μl of the dispersed sensitized colloidal gold solution was taken and the optical density at 520 nm was measured.
j) The optical density at 520 nm was adjusted to 6.0 with colloidal gold storage buffer.
k) The reagent was stored at 4ºC until its use.

Streptoavidin was dissolved in ultrapure water to a concentration of 100 μg/ml, and the same procedure was performed.

2) Fabrication of conjugate pad.

a) The anti-human CRP (Anti-h CRP 6407 SPTN-5)-sensitized colloidal gold solution and the streptoavidin-sensitized colloidal gold were separated using an ultrasonic cleaner.
b) Each colloidal gold solution, ultrapure water, and gold colloid coating buffer were mixed in a 2 ml tube.
c) The entire amount was applied evenly onto a glass fiber pad.
d) The pad was placed in a desiccator and dried under reduced pressure for more than one day.
e) The pad was stored in a refrigerator until its use.

3) Preparation of biotinylated BSA.

a) BSA was dissolved to 5 mg/ml with carbonate buffer so that the final volume was 1,000 μl.
b) The biotinylation reagent was adjusted to 40 mg/ml with ultrapure water so that the final volume was at least 12.5 μl.
c) 12.5 μl of the biotin reagent solution prepared in b) was added to 1,000 μl of the BSA

solution prepared in a).

d) The mixed solution was allowed to stand at room temperature for 2 h.

e) Glycine was adjusted to 10 mg/ml with ultrapure water so that the final liquid volume was 100 μl or more.

f) 100 μl of the glycine solution prepared in e) was added to the tube in d).

g) The solution was replaced with PBS.

h) After measuring the optical density at 280 nm of the internal solution, it was adjusted with PBS to 1 mg/ml and stored in a refrigerator until its use.

4) Preparation of antibody-immobilized membrane.

a) Anti-human CRP antibody (Goat anti-human CRP antibody) was replaced with PBS and adjusted to two different concentrations.

b) 100 μl of the anti-CRP polyclonal antibody as test line 1 and 2, and the biotin-labelled BSA (1.0 mg/ml) as a control line were prepared.

c) The antibodies were applied to a membrane using a coating machine.

d) The membrane was dried at 37ºC for 1 h.

e) The entire membrane was immersed in blocking buffer, and it was allowed to stand at room temperature for 30 min.

f) The membrane was transferred to the washing/stabilizing buffer for 30 min at room temperature while stirring.

g) It was dried at room temperature for more than one day and night.

h) It was stored in a refrigerator protected from the light.

5) Assembly of immunochromato strip.

a) The antibody-immobilized membrane was placed at a position of 20 mm from the bottom edge of the backing sheet.

b) The sample absorbent pad was aligned with the top edge of the backing sheet and pasted.

c) A plasma separation pad was attached at 17 mm from the bottom edge of the backing sheet.

d) A conjugate pad was pasted 11 mm from the bottom edge of the backing sheet.

e) Align the sample pad with the bottom edge of the backing sheet and paste it.

f) The sheet was cut to 5 mm width using a cutting module.

g) The sheet was put in the housing case.
