# Supplementary material for: Preparing of Point-of-Care Reagents for Risk Assessment in the Elderly at Home by a Home-Visit Nurse and Verification of Their Analytical Accuracy
Source: Diagnostics (Basel). 2023 Jul 19;13(14):2407. doi: 10.3390/diagnostics13142407 (PMC10378029; doi:10.3390/diagnostics13142407)
Supplement: Supplementary file 1 [file diagnostics-13-02407-s001.zip › Supplementary File S3.pdf]

Supplementary file S3. The characteristics of patients.

Patient No.	Sex	Age	Diagnosis	Outcome	Medical history	Clinical bacteriological examination		
						Blood culture	Suction sputum or expectorated sputum	Urinary catheter
1	M	68	Legionella pneumonia	Hospital transfer	Hypertension, Dyslipidemia, Mental retardation	-	Neisseria α-streptococcus 1+, Corynebacterium sp. 2+, Coagulase(-) Staphylococcus 2+	Escherichia coli 3+
2	F	82	Bacterial pneumonia	Hospital transfer	Mycobacterium avium complex	-	Neisseria α-streptococcus 1+	-
3	M	75	Aspiration pneumonia	Healing	UNK	-	Neisseria α-streptococcus 1+, Candida albicans 1+, Coagulase(-) Staphylococcus 1+	-
4	M	82	Aspiration pneumonia	Healing	Rectal cancer	-	Neisseria α-streptococcus 3+, Hamophilus influenzae 3+	-
5	M	70	Bacterial pneumonia	Hospital transfer	Cerebral infarction, Subarachnoid hemorrhage	-	-	-
6	F	79	Atypical pneumonia	Healing	Non-tuberculos mycobacterial disease, Chronic bronchitis	-	Mycobacterium avium, Aspergillus niger 1+, Candida albicans 1+, Neisseria α-streptococcus 3+	-
7	M	31	Bacterial	Healing	UNK	-	Neisseria α-streptococcus 2+	-

			pneumonia					
8	F	82	Bacterial pneumonia	Healing	Cardiomyopathy, Alzheimer dementia	-	Candida albicans 1+, Neisseria α-streptococcus 1+	-
9	F	94	Bacterial pneumonia	Mortality	Hypertension, Atrial fibrillation, Pulmonary edema	-	Candida albicans 1+, Neisseria α-streptococcus 3+, Coagulase(-) Staphylococcus 1+	-
10	F	72	Aspiration pneumonia	Healing	Septic shock, Acute respiratory failure, Acute heart failure	-	Candida albicans 1+, Pseudomonas aeruginosa 3+, Corynebacteirum sp. 2+	Enterococcus faecium 1+
11	M	61	Bacterial pneumonia	Healing	Hypertension, Atrial fibrillation, Cerebral infarction	-	-	-
12	M	70	Bacterial pneumonia	Mortality	Hypertension, Lung abscess, Acute kidney injury, Hypocalcaemia, Mental illness	-	Pseudomonas aeruginosa 3+, Neisseria α-streptococcus 3+, Stenotrophomonas maltophilia 1+, Mycobacterium intracellulare	-
13	M	74	Aspiration pneumonia	Healing	Respiratory failure, Pneumothorax, Disturbance of consciousness,	-	Neisseria α-streptococcus 2+, Coagulase(-) Staphylococcus 2+	-

					Dysphagia			
14	M	82	Bacterial pneumonia	Hospital transfer	Cerebral infarction,<br>Diabete,<br>Brain tumor,<br>Lung cancer	-	Neisseria α-streptococcus 2+<br><Alveolar lavage fluid><br>MRSA 1+,<br>Enterococcus faecium 1+,<br>Candida albicans 1+	-
15	M	75	Pneumonia	Healing	Diabetes,<br>Hypertension,<br>Dyslipidemia	-	Neisseria α-streptococcus 3+	-
16	M	73	Pneumonia	Healing	Hypertension,<br>Chronic renal failure,<br>Hyperuricemia	-	Neisseria α-streptococcus 3+	-
17	F	76	Bacterial pneumonia	Healing	Lung cancer,<br>Hypertension,<br>Combined pulmonary fibrosis and emphysema,<br>Dyslipidemia,<br>Chronic obstructive pulmonary disease	-	Candida albicans 1+,<br>Neisseria α-streptococcus 2+,<br>Bifidobacterium sp. 1+	-
18	F	79	Aspiration pneumonia	Hospital transfer	Parkinson’s disease	-	Enterobacter aerogenes 1+,<br>Candida albicans 1+,<br>Neisseria α-streptococcus 2+	Escherichia coli 3+,<br>γ-Streptococcus 3+

19	F	62	Bacterial pneumonia	Healing	Chronic obstructive pulmonary disease, Essential thrombocythemia	-	Pseudomonas aeriginosa 1+, Neisseria α-streptococcus 2+	-
20	M	52	Aspiration pneumonia	Mortality	Bone marrow tumor, Malignant glioma, Dysphagia		MSSA, Neisseria α-streptococcus 3+, Klebsiella pneumonia 1+	-
21	F	73	Aspiration pneumonia	Hospital transfer	Symptomatic epilepsy Pulmonary edema	-	Neisseria α-streptococcus 2+	-
22	M	73	Aspiration pneumonia	Hospital transfer	Spastic paralysis Iron deficiency anemia	-	MRSA 2+, Corynebacterium sp. 2+, Escherichia coli 3+, Aspergillus fumigatus 1+, Neisseria α-streptococcus 1+	-
23	F	23	Suspected aspiration pneumonia	Hospital transfer	Right empyema, Respiratory failure, Disuse syndrome	Pseudo monas aerugin osa Mtype	Pseudomonas aeruginosa Mtype 2+, Neisseria α-streptococcus 2+, Enterococcus faecalis 2+	-
24	M	76	Chronic obstructive pulmonary disease	Healing	Pneumonia, Atrial fibrillation, Heart failure	-	-	-
25	M	84	Aspiration	Healing	Dysphagia,	-	Enterobacter cloacae 2+,	-

			pneumonia		Xerostomia		Neisseria α-streptococcus 3+	
26	F	48	Streptococcal sepsis	Healing	UNK	-	Pseudomonas aeruginosa 1+, Klebsiella pneumoniae 1+, Coagulase(-) Staphylococcus 2+, Candida albicans 2+	-
27	F	55	Influenzal pneumonia	Healing	UNK	-	Coagulase(-) Staphylococcus 1+, γ-Streptococcus 1+	-
28	M	81	Influenzal pneumonia	Healing	UNK	-	Klebsiella pneumoniae 1+, Candida tropicalis 2+, Neisseria α-streptococcus 3+	-
29	F	89	Aspiration pneumonia	Healing	Cerebral infarction, Dementia, Osteoporosis	-	Streptococcus dysgalactiae 3+, Klebsiella pneumoniae 1+, Neisseria α-streptococcus 3+, Coagulase(-) Staphylococcus 2+, Candida albicans 1+, Neisseria α-streptococcus 1+	-
30	M	74	Staphylococcal pneumonia, Aspiration pneumonia	Mortality	UNK	MSSA	MSSA 3+, Candida krusei 2+, Neisseria α-streptococcus 3+	-

31	M	66	Bacterial pneumonia	Healing	Chronic hepatitis, Enteritis	-	Neisseria α-streptococcus 2+	-
Mean±SD		72.0 ± 12.6						

M: Male; F: Female; UNK: Unknown
