# Supplementary figures and images for: Preparing of Point-of-Care Reagents for Risk Assessment in the Elderly at Home by a Home-Visit Nurse and Verification of Their Analytical Accuracy
Source: Diagnostics (Basel). 2023 Jul 19;13(14):2407. doi: 10.3390/diagnostics13142407 (PMC10378029; doi:10.3390/diagnostics13142407)

File S4. Representative images of Actim CRP at each detection range.

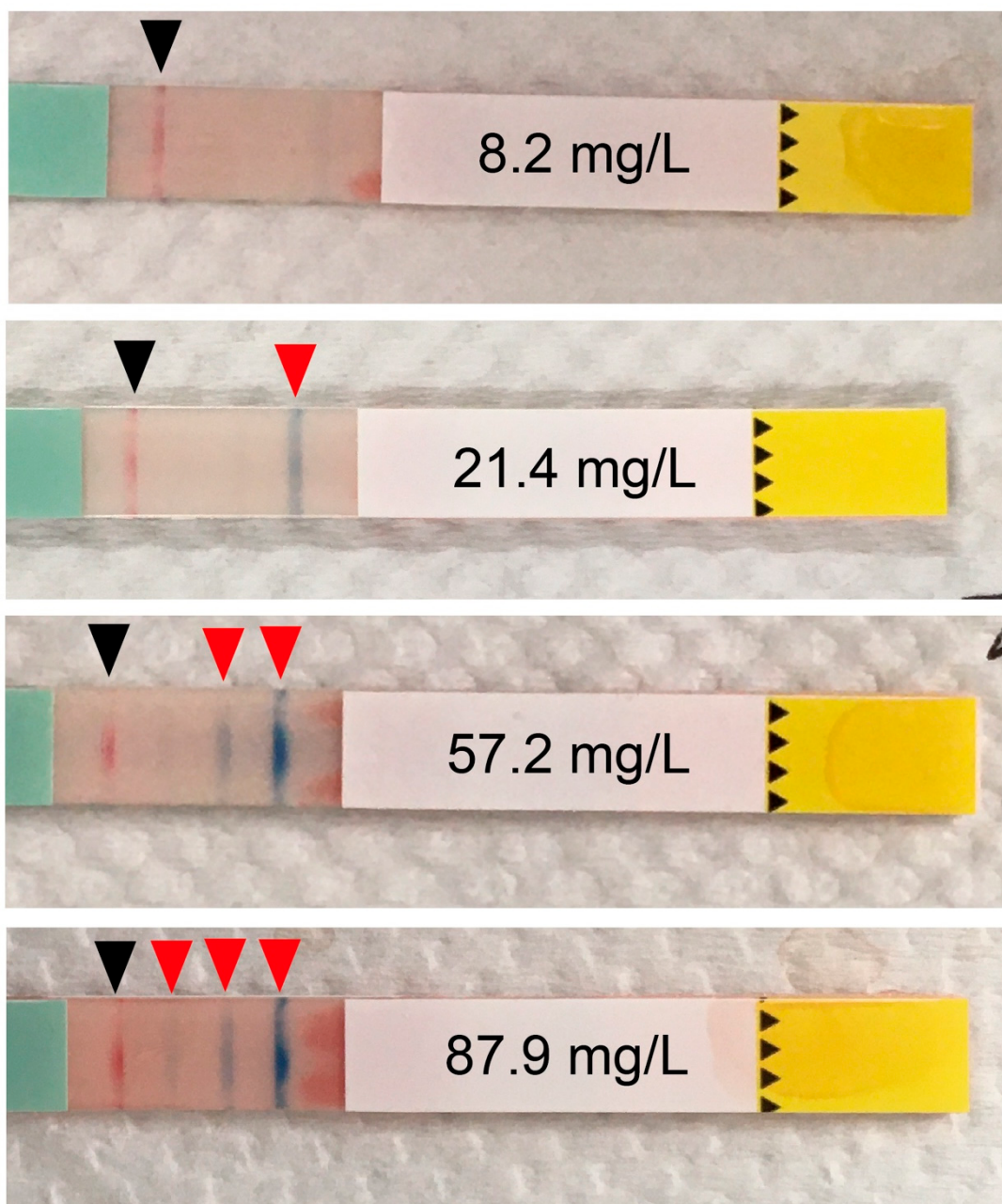

Supplement: Supplementary file 1 [file diagnostics-13-02407-s001.zip › Supplementary File S4.pdf]
